# Cytokine Expression and Macrophage Localization in Xenograft and Allograft Tumor Models Stimulated with Lipopolysaccharide

**DOI:** 10.3390/ijms19041261

**Published:** 2018-04-23

**Authors:** Junko Masuda, Tsukasa Shigehiro, Takuma Matsumoto, Ayano Satoh, Akifumi Mizutani, Chiho Umemura, Shoki Saito, Mayumi Kijihira, Eiji Takayama, Akimasa Seno, Hiroshi Murakami, Masaharu Seno

**Affiliations:** 1 Department of Medical Bioengineering, Graduate School of Natural Science and Technology, Okayama University, Okayama 700-8530, Japan; en20727@s.okayama-u.ac.jp (T.S.); pvxn4t5n@s.okayama-u.ac.jp (T.M.); ayano113@cc.okayama-u.ac.jp (A.S.); mizut-a@cc.okayama-u.ac.jp (A.M.); pmyu2alv@s.okayama-u.ac.jp (C.U.); p4hi0alp@s.okayama-u.ac.jp (S.S.); aseno@okayama-u.ac.jp (A.S.); muraka-h@cc.okayama-u.ac.jp (H.M.); mseno@okayama-u.ac.jp (M.S.); 2 Department of Applied Chemistry and Biotechnology, Faculty of Engineering, Okayama University, Okayama 700-8530, Japan; pvc15we6@s.okayama-u.ac.jp; 3 Department of Oral Biochemistry, School of Dentistry, Asahi University, Gifu 501-0223, Japan; takayama@dent.asahi-u.ac.jp

**Keywords:** myeloid-derived suppressor cells (MDSCs), dendritic cells (DCs), M1 macrophages, M2 macrophages, xenograft tumor, allograft tumor, lipopolysaccharide (LPS)

## Abstract

T cell-deficient mice such as nude mice are often used to generate tumor xenograft for the development of anticancer agents. However, the functionality of the other immune cells including macrophages, dendritic cells (DCs), and myeloid-derived suppressor cells (MDSCs) in the xenograft are largely unknown. Macrophages and dendritic cells (DCs) acquire functionally distinct properties in response to various environmental stimuli; the interaction of these cells with MDSCs in tumor microenvironments regulates cancer progression. Nude mice are less likely to reject human cancer cells because of major histocompatibility complex (MHC) mismatches. The tumor microenvironment in a xenograft, comprising human and mouse cells, exhibits more complex bidirectional signaling and function than that of allograft. Here, we evaluated the differences of myeloid cells between them. Plasma interferon-γ and interleukin-18 concentrations in the xenograft tumor model after lipopolysaccharide (LPS) administration were significantly higher than those in the allograft tumor model. MHC class I, II, and CD80 expression levels were increased in CD11b^+^ and MDSC populations after LPS administration in the spleen of a xenograft tumor model but not in that of an allograft tumor model. Additionally, the number of CD80- and mannose receptor C type 1 (MRC1)-expressing cells was decreased upon LPS administration in the tumor of the xenograft tumor. These results suggest that functions of macrophages and DCs are sustained in the xenograft, whereas their functions in response to LPS were suppressed in the allograft. The findings will encourage the consideration of the effects of myeloid cells in the xenograft for drug development.

## 1. Introduction

Many human cancer cell lines derived from patients have been established and studied over the past 40 years. In this cancer research, the transplantation of xenograft of the cancer cell lines into immunodeficient mice is commonly used to produce in vivo preclinical models for drug evaluation, biomarker identification, biologic studies, and personalized medicine strategies [1]. Because T cells mainly contribute xenograft rejection, immunodeficient mice lacking T cells, known as nude mice, are frequently used for human cancer research. These mice have myeloid cells, such as macrophages, myeloid-derived suppressor cells (MDSCs), and dendritic cells (DCs), which regulate cancer progression [2]. Understanding the functions of the myeloid lineage cells in the xenograft is necessary to precisely predict patient outcomes in the drug development.

Macrophages can be mainly classified into two phenotypes, M1 and M2 macrophage. These phenotypes are regulated by the microenvironment. The M1 macrophages show a pro-inflammatory and cytotoxic phenotype and are activated by the gram-negative bacterial endotoxin lipopolysaccharide (LPS) or pro-inflammatory cytokines such as interferon-γ (IFN-γ) and tumor necrosis factor-α (TNF-α) [3]. Thus, M1 macrophages have the potential to eliminate tumor cells. In contrast, M2 macrophages are activated by anti-inflammatory cytokines such as interleukin (IL)-4 and IL-13 and express high levels of mannose receptor C-type 1 (MRC1). M2 macrophages are thought to be synonymous with tumor-associated macrophages (TAM) because they modulate inflammatory responses by decreasing responsiveness to toll-like receptors (TLRs) and IFN-γ [2,4,5,6,7].

MDSCs represent a heterogeneous cell population whose members share a capacity to exert a suppressor function [8,9,10] and whose numbers increase with the growth of human and murine cancers [11,12,13,14,15,16]. Although MDSCs are characterized by the dual expression of Gr-1 and CD11b in mice and are regarded as a distinct population from M2 macrophages, they share many characteristics in terms of mechanisms that sustain and promote tumor growth [2].

The experimental xenogeneic tumor microenvironment generated in immunodeficient mice consists of mouse stromal cells and these myeloid cells. However, the commonalities of the distributions and functions of M1/M2 macrophages, DCs, and MDSCs between xenograft and allograft tumors are still controversial. In this study, we examined the difference of functions of myeloid lineage cells in between xenografts and allografts using human and mouse collocate cancer cells implanted in nude mice.

## 2. Results

### 2.1. Expression of Immune Cells in Nude Mice

**Figure 1:**
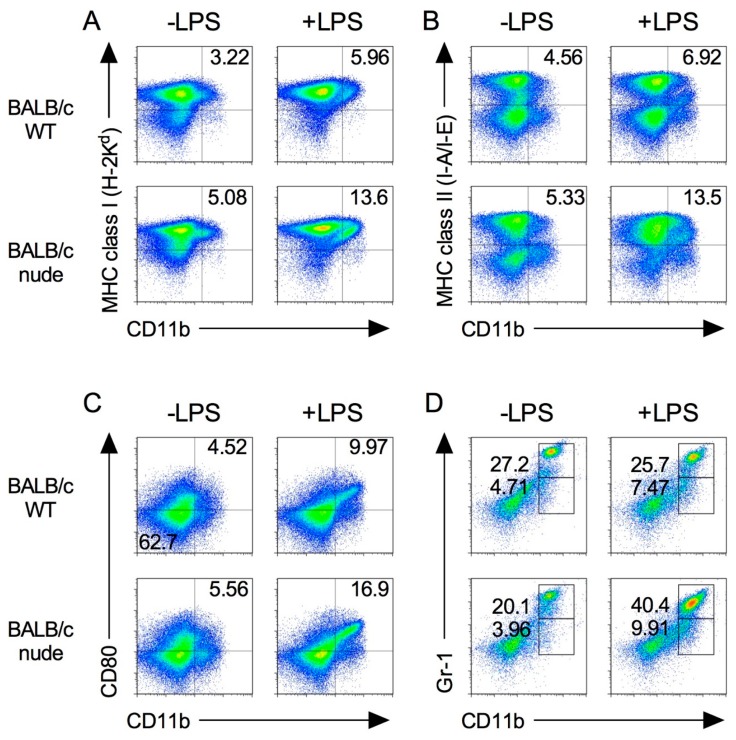
Expression of immune cells in BALB/c-nude mice. Splenocytes were isolated from spleen from BALB/c and BALB/c-nude mice after 24 h from i.p. injection of 500 μg LPS (+LPS) or PBS control (−LPS). The expression level of MHC class I (**A**), class II (**B**), CD80 (**C**), and Gr-1 (**D**) on CD11b^+^ cells was analyzed by flow cytometry. Results shown are representative of at least three independent experiments.

We first characterized the population of immune cells in the spleens of nude mice in which both CD4^+^ and CD8^+^ T cells were absent. As shown in Figure 1, major histocompatibility complex (MHC) class I and II molecules and CD80, which are required for T cell activation, are highly expressed in CD11b^+^ M1 macrophage [17,18]. Splenic MHC class I expression on CD11b^+^ cells increased following LPS stimulation in both BALB/c wild type (WT) and BLAB/c-nude mice (Figure 1A). The trend was also observed in MHC class II and CD80 expression on CD11b^+^ cells (Figure 1B,C).

In addition, MDSCs were identified by the co-expression of the myeloid lineage differentiation antigens Gr-1 and CD11b [16,19]. The percentages of Gr-1^dim^ CD11b^+^ and Gr-1^hi^ CD11b^+^ MDSCs in nude mice were higher than those in WT mice (Figure 1D).

We next examined LPS concentration to stimulate nude mice. Although 50 μg LPS injection is enough for WT mice to produce IFN-γ [20], 500 μg of LPS was needed for nude mice (Figure S1). Thus, we decided to stimulate nude mice with 500 μg of LPS for analysis in the subsequent experiment.

We further analyzed the phenotype of macrophage and MDSC populations after LPS stimulation via intraperitoneal (i.p.) in nude mice. As shown in Figure 1, the expression of MHC class I, II and CD80 on CD11b^+^ cells and MDSCs increased in nude mice by LPS. These results suggest that M1 macrophages and MDSCs are functional in nude mice.

### 2.2. Pro-Inflammatory Cytokine Production in Nude Mice Stimulated by LPS

**Figure 2:**
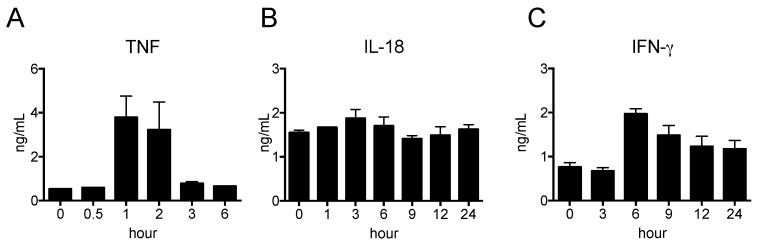
Pro-inflammatory cytokine productions in nude mice stimulated by LPS. LPS was i.p. injected and blood samplings were carried out at indicated time-points. The serum levels of TNF (**A**), IFN-γ (**B**) and IL-18 (**C**) were determined by ELISA. The data are presented as the mean ± SEM. *n* = 5.

The pro-inflammatory cytokines TNF, IL-12, and IL-18 are mainly produced by macrophages and DCs, whereas IFN-γ is produced by natural killer (NK) cells. We determined the kinetics of the cytokine levels in peripheral blood after LPS injection. As shown in Figure 2A, the serum level of TNF was the highest at 1 h and decreased to basal levels at 3 h, whereas that of IFN-γ was the highest at 6 h and then gradually decreased (Figure 2B). In contrast, the serum level of IL-18 did not change over the course of 24 h after LPS injection (Figure 2C). The serum levels of IL-1β, IL-10 and IL-12 were undetectable by ELISA. Thus, we decided to use peripheral blood collected at 1 h (for TNF), and 1, 3, 6, 9, 12, and 24 h (for IFN-γ and IL-18) after LPS injection for analysis in the subsequent experiment.

### 2.3. Serum Pro-Inflammatory Cytokine Levels in the Xenograft Tumor Model after LPS Stimulation

**Figure 3:**
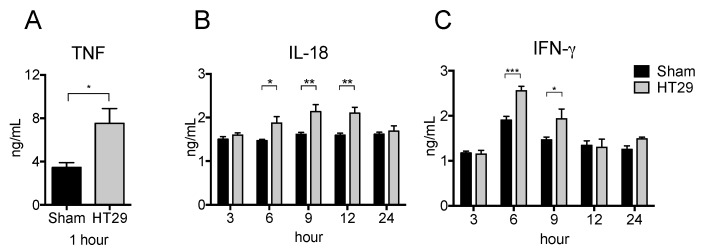
Increased serum pro-inflammatory cytokine levels in xenograft tumor mice after LPS stimulation. HT29 cells or PBS were s.c. injected into BALB/c-nu/nu mouse. On the day 21 after tumor transplantation, 500 μg LPS was i.p. injected into the xenograft mice and peripheral blood was collected at indicated hour. The serum levels of TNF (**A**), IFN-γ (**B**), and IL-18 (**C**) were detected by ELISA. The data are presented as the mean ± SEM assessed by a Student’s two-tailed *t*-test. * *p* < 0.05; ** *p* < 0.01; *** *p* < 0.001. *n* = 5.

To examine the respective cytokine production in the xenograft tumor model, we subcutaneously (s.c.) injected human colon adenocarcinoma cell lines (HT29) or PBS (as a control) into nude mice and then i.p. injected LPS at 21 d after inoculation. As shown in Figure 3A,C, the serum levels of TNF and IFN-γ significantly increased in HT29 tumor-bearing mice at 1 h and at 3–6 h, respectively. Furthermore, the serum level of IL-18 was also significantly increased in HT29 tumor bearing mice at 6–12 h (Figure 3B). These results suggest that HT29 tumor-bearing mice have more potential to promote pro-inflammatory responses than sham control mice.

### 2.4. Serum Pro-Inflammatory Cytokine Levels in the Allograft Tumor Model Were Unchanged Even after LPS Stimulation

**Figure 4:**
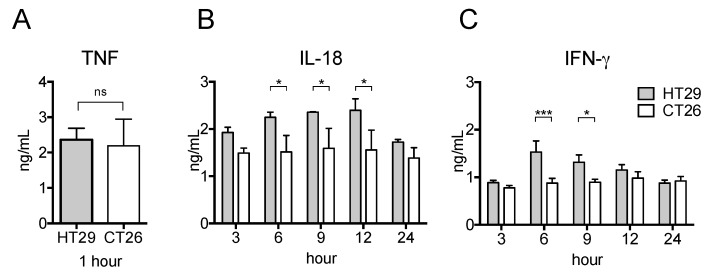
Serum pro-inflammatory cytokine levels in xenograft and allograft tumor models after LPS stimulation. HT29 cells or CT26 cells were s.c. transplanted into BALB/c-nu/nu mice. On the day 21 after transplantation, 500 μg LPS was i.p. injected and peripheral blood was collected at indicated hour. The serum levels of TNF (**A**), IFN-γ (**B**), and IL-18 (**C**) were detected by ELISA. The data are presented as the mean ± SEM assessed by a Student’s two-tailed *t*-test. * *p* < 0.05; *** *p* < 0.001; ns: not significant. *n* = 5.

Given the increased production of pro-inflammatory cytokines in HT29 tumor-bearing nude mice, we next studied the respective cytokine production in the xenograft and allograft tumor models after LPS treatment. To this end, we s.c. injected human or mouse colon adenocarcinoma cell lines (HT29 or CT26, respectively) in nude mice. Interestingly, as shown in Figure 4B,C, the serum levels of IL-18 and IFN-γ of CT26 tumor-bearing mice did not increase whereas the serum level of TNF was as high as HT29 tumor-bearing mice (Figure 4A). Thus, the potential for pro-inflammatory responses in the xenograft tumor model is much greater than in the allograft model.

### 2.5. Splenic M1 Macrophages and MDSCs Increase after LPS Stimulation in the Xenograft Tumor Model

**Figure 5:**
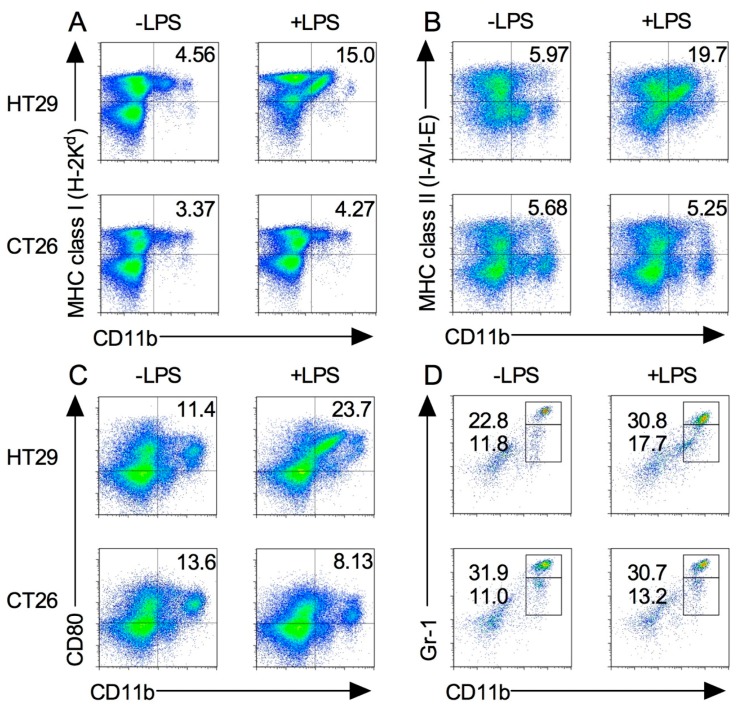
M1 macrophages and MDSCs in xenograft and allograft tumor models after LPS stimulation. HT29 and CT26 tumor-bearing mice were treated with LPS on the day 21 from tumor transplantation and splenocytes were isolated at 24 h. The expression levels of MHC class I (**A**), class II (**B**), CD80 (**C**), and Gr-1 (**D**) on CD11b^+^ cells were analyzed by flow cytometry. Results are shown are representative of at least three independent experiments.

The spleen traps pathogens and antigens in circulating blood and initiates innate and adaptive immune responses. Macrophages exist in the spleen in a steady state, whereas MDSCs migrate there during inflammation or cancer progression [16,18]. To determine the differences in the expression and functions of M1 macrophages and MDSC subsets, HT29 and CT26 tumor-bearing mice were administered with LPS as described above, and their splenocytes were stained with the marker of M1 macrophage. As shown in Figure 5A–C, MHC class I, II, and CD80 expression levels on CD11b^+^ cells were increased in HT29 tumor-bearing mice following LPS injection, whereas MHC class I and II expression levels on CD11b^+^ cells were not changed in CT26 tumor-bearing mice following LPS injection. Notably, LPS injection decreased CD80 expression on CD11b^+^ cells in CT26 tumor-bearing mice (Figure 5A–C).

We next examined the MDSC population in spleen. Compared with the steady state (Figure 1D), the percentage of MDSCs in HT29 tumor-bearing mice slightly increased during tumor growth and even further after LPS injection (Figure 5D). In contrast, the percentage of the MDSC population in CT26 tumor-bearing mice was dramatically increased during tumor growth, and no difference of the population was observed following LPS injection (Figure 5D). Taken together, these results indicate that the functions of these splenic myeloid cells are different between xenograft and allograft tumor models.

### 2.6. Expression of Tumor-Infiltrated M1 and M2 Macrophages before and after Induction by LPS

**Figure 6:**
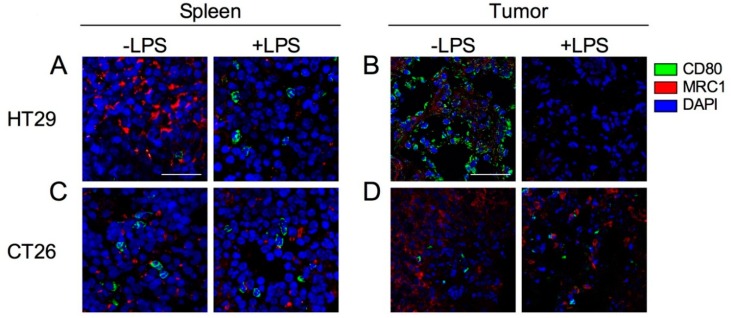
Confocal images of CD80 and MRC1 expression cells in spleen and tumor with or without injection of LPS. HT29 and CT26 tumor-bearing mice were treated with LPS (+LPS) or PBS control (−LPS) on the day 21 from tumor transplantation and tumor and spleen were collected after 24 h from LPS treatment. (**A**) Spleen in HT29 tumor-bearing mice. (**B**) Tumor in HT29 tumor-bearing mice. (**C**) Spleen in CT26 tumor-bearing mice. (**D**) Tumor in CT26 tumor-bearing mice. CD80, MRC1, and cell nuclei (DAPI) are represented in green, red, and blue, respectively. Representative images from at least three independent experiments are shown. Each scale bar indicates 50 μm.

M1 macrophages and activated DCs express CD80, whereas M2 macrophages express MRC1. Additionally, CD80 is also expressed on activated DCs. To investigate the localization and function of M1/M2 macrophages in the spleens and tumors of HT29 and CT26 tumor-bearing mice, we stained spleen and tumor sections with M1 and M2 macrophage markers, CD80 and MRC1, respectively. In the spleens of HT29 tumor-bearing mice, CD80-expressing cells were very few and MRC1-expressing cells were dominant at steady state (Figure 6A). Following exposure to LPS, the number of CD80-expressing cells were increased, whereas that of MRC1-expressing cells were decreased (Figure 6A). In tumor of HT29 tumor-bearing mice, CD80-expressing-cells predominantly localized with tumor cells, and MRC1-expressing cells were also present at steady state (Figure 6B). Following exposure to LPS, the number of CD80- and MRC1-expressing cells were decreased (Figure 6B). In the spleens of CT26 tumor-bearing mice, CD80- and MRC1-expressing cells were present but they did not increase following exposure to LPS (Figure 6C). Notably, very few CD80-expressing cells and numerous MRC1-expressing cells were observed in the CT26 tumor compared with the HT29 tumor, and the populations did not change following LPS exposure (Figure 6D). Taken together, these results suggest that the physiological function of CD80-expressing cells after LPS signaling is retained in the xenograft tumor model but not the allograft tumor model.

## 3. Discussion

Macrophages and MDSCs play an important role in systemic and tumor immunity. Thus, using patient-derived tumor cell lines in xenograft experiments has contributed to our understanding of the mechanisms of macrophage infiltration into tumors [21]. However, the xenograft tumor microenvironment is composed of human and murine cells, and these different species might result in more complicated bidirectional signaling and function in the stromal cells compared with syngeneic tumor microenvironments. In this study, we attempted to clarify the difference with respect to M1/M2 macrophages, DCs, and MDSCs between xenograft and allograft tumor models and found that the xenograft tumor model produces more pro-inflammatory cytokines than the allograft tumor model.

After being primed by LPS, macrophages and DCs become activated through TLR4-mediated cytokine production (TNF, IL-12, and IL-18) and have increased surface expression of several stimulating ligands such as MHC molecules and CD80, leading to the activation of NK cells which produce IFN-γ [22]. More recently, it has been reported that LPS directly stimulates IFN-γ production in NK cells mediated by the established TLR4-mediated signaling pathway [23]. The LPS injections we utilized in this study were an appropriate way to probe macrophage and DC function in the tumor models because LPS drives macrophages to a preferentially M1 phenotype [24,25].

LPS i.p. administration is rapidly absorbed into the systemic circulation [26] and reaches other organs, including tumors, and is trapped in the spleen in which macrophages and DCs are accommodated [18,27]. Similarly, when tumor antigens are produced during tumor cell apoptosis, the antigens reach the systemic circulation and are eventually filtered through the spleen. Interestingly, we found that the serum levels of IL-18 and IFN-γ in xenograft tumor model mice after LPS stimulation were significantly increased compared with sham mice (Figure 4) and allograft model mice (Figure 5). Our study also showed that the pro-inflammatory states in the spleen and the tumor of xenograft and allograft tumor models were completely different even before LPS administration (Figure 6). These results indicate that the production of pro-inflammatory cytokines by M1 macrophages in the xenograft tumor models are elevated even when T cells are absent and phagocytotic activity of macrophages was adequately induced in the tumor environment of the xenograft tumor model, whereas macrophage-mediated tumor destruction was inhibited and a splenic chronic inflammation-like state was induced in the allograft tumor model [28,29,30]. One of the reasons of this may be MDSCs that were not induced by cancer growth in the xenograft model but were induced in the allograft model (Figure 5). MDSCs, TAMs, and some DC populations suppress immune activity, which is enhanced by their interactions with each other [24]. Although an increase of MDSCs occurred in the allograft tumor model, it is quite interesting that MDSCs did not increase during tumor growth in the xenograft tumor model. Since G-CSF and GM-CSF convert myeloid progenitor cells to MDSCs, only the allograft model that expressed mouse C-CSF/GM-CSF might exhibit increasing MDSC population. The other possibility for this is that MDSCs are derived from NK cells. Human tumor cells do not express mouse MHC class I molecules that are recognized by mouse immune cells such as NK cells [31]. Although NK cells can be converted into MDSCs in allograft tumor models, this conversion is inhibited by the presence of IL-2 [32]. These reports suggest that an unknown mechanism of cell-to-cell contact involving MHC-dependent signaling is required to regulate MDSCs in systemic and tumor microenvironments and that these mechanisms might induce different immune responses in xenograft and allograft tumor models [25,33].

Our study showed that MRC1 expression in the spleen and the tumor was repressed after LPS injection in the xenograft tumor model but did not change in the allograft tumor model (Figure 6). IFN-γ suppresses the expression of genes that are required in M2 macrophages, following a decrease in MRC1 expression levels [34]. In addition to the plasticity between M1 and M2 macrophage phenotypes, fully polarized M2 macrophages can be converted into M1 macrophages following stimulation by LPS [35]. Our results indicate that the functions of M1 and M2 macrophages are proper in the xenograft tumor model, whereas the function of both is suppressed in the allograft tumor model.

In conclusion, the research has found that the functions of macrophages and MDSCs in xenograft tumor models were different from the allograft tumor model. The underlining mechanism in xenograft tumor models might involve chronic activation of M1 macrophages leading to the inhibition of MDSC expression. Cancer research using xenograft tumor models is important for investigation of cancer therapies. However, these results are not always consistent with clinical outcomes [1]. Our results imply that one of the reasons for this might be that host immune responses generated in systemic and tumor microenvironments in xenograft models do not represent complex tumorous immune systems compared with spontaneous tumors or allograft tumor implantation. In addition, research focusing on the tumor microenvironment created by the presence of mouse stromal cells might also be necessary to fully understand the influence of cell-to-cell contact from different species. Therefore, in vivo cancer studies with xenograft tumor model should be more carefully interpreted to precisely predict patient outcomes of candidates as an anticancer drug.

## 4. Materials and Methods

### 4.1. Reagents and Antibodies (Abs)

LPS (Lot. No. L8274) was purchased from Sigma-Aldrich (St. Louis, MO, USA). Anti-mouse CD11b (M1/70) and MHC Class I (H2K^d^, SF1-1.1) monoclonal (m) Abs were purchased from BD Biosciences (San Jose, CA, USA). Anti-mouse CD16/CD32 (93), and Gr-1 (RB6-8C5) mAbs were purchased from eBioscience (San Diego, CA, USA). Anti-CD11c (N418), CD80 (16-10A1), MHC Class II (I-A/I-E, M5/114.15.2) and 7-amino-actinomycin D (7AAD) from Tonbo Biosciences (San Diego, CA, USA). Rabbit anti-MRC1 polyclonal (p) Ab was from Bioss Antibodies (Woburn, MA, USA). Alexa 568-conjugated goat anti-rabbit IgG was from Molecular Probes (Eugene, OR, USA).

### 4.2. Animals

Female BALB/c (WT) and BALB/c nu/nu (nude) mice (age, 4 weeks; weight 13–17 g) were obtained from Charles River Inc. (Kanagawa, Japan) and maintained under specific pathogen-free conditions at the Tsushima-kita Branch, Department of Animal Resources, Advanced Research Center, Okayama University. Animals were maintained at 22–26 °C and 50% humidity with a 12-h light/dark cycle and were fed a standardized diet and had ad libitum access to autoclaved tap water. All animal experiments were reviewed and approved by the ethics committee (Animal Care and Use Committee) for animal experiments of Okayama University under the project identification code IDs OKU-2015229 (12 May 2015), OKU-2016225 (6 June 2016) and OKU-2016361 (21 September 2016).

### 4.3. LPS Injection

A total of 500 μg (for BALB/c) or a serial concentration (for BALB/c nu/nu) of LPS (in 200 μL PBS/body) was intraperitoneally (i.p.) injected and peripheral blood and tissue samplings were obtained within 24 h.

### 4.4. Tumor Cell Cultures

Colon adenocarcinoma cell lines, HT29 and CT26 were purchased from the American Type Culture Collection (Rockville, MD, USA) and maintained in Roswell Park Memorial Institute (RPMI) 1640 medium (Sigma-Aldrich, St. Louis, MO, USA) supplemented with 10% (*v*/*v*) heat-inactivated fetal bovine serum (FBS) (SAFC Biosciences, Lenexa, KS, USA) and 1% (*v*/*v*) antibiotic-antimycotic solution (10,000 U/mL penicillin, 10,000 μg/mL streptomycin, and 25 μg/mL amphotericin B; Life Technologies, Gaithersburg, MD, USA). The cultures were incubated in a humidified atmosphere with 5% CO_2_ at 37 °C.

### 4.5. Tumor Cell Implantations

HT29 cells (3 × 10^6^ cells/body) or CT26 cells (1 × 10^5^ cells/body) in 200 μL PBS were s.c. implanted into the right flank of a 5-week-old female mouse as according to a previously study [16,36]. A sham mouse, as control experiments, was injected PBS alone. On the day 21, 500 μg LPS in 200 μL PBS was i.p. injected and peripheral blood and tissue samplings were obtained within 24 h.

### 4.6. Assays for Cytokine Levels

Mouse peripheral blood was centrifuged at 500× *g* for 5 min. After clear plasma was collected, TNF, IFN-γ, and IL-18 levels were evaluated by using cytokine-specific enzyme-linked immunosorbent assay (ELISA) kits. The mouse TNF and IFN-γ assay kits were from BD Biosciences. The mouse IL-18 assay kit was from BMN (Nagoya, Japan).

### 4.7. Flow Cytometry

Splenocytes were isolated as according to a previously study [37]. Splenocytes (2 × 10^6^) were incubated with anti-CD16/CD32 mAb (5 μg/mL) for 20 min on ice then stained with anti-CD4 and CD8α, H2K^d^, I-A/I-E, Gr-1 and CD11b Abs (1 μg/mL) for 30 min on ice and washed with 2% FBS, 1 mM EDTA and 0.1% sodium azide in PBS followed by labelling with 7AAD (1 μg/mL). The stained cells were analyzed by Accuri™ (BD Biosciences) and FlowJo Software (Treestar, Inc., San Carlos, CA, USA).

### 4.8. Immunohistochemical Analyses

Tissues were placed in OTC compound (Miles Laboratories, Naperville, IL, USA) and quickly frozen in liquid nitrogen and stored at −80 °C. Frozen sections (6 μm) were fixed with 4% formaldehyde neutral buffer solution (Nacalai Tesque, Kyoto, Japan) for 15 min followed by permeabilization with 0.1% Triton X-100 in PBS for 5 min and blocked with 4% BSA in PBS for 15 min at room temperature. The sections were then incubated with biotinylated FITC-labelled anti-MHC-Class II (5 μg/mL) or anti-MRC1 Abs (1:50 dilution) for 1 h. After washing in PBS, the sections were incubated with secondary antibodies (1:50 dilution) for 15 min. Sections were mounted with Vectashield**^®^** mounting with DAPI (Vector Laboratories, Burlingame, CA). The immunofluorescence images visualized under a confocal microscope (FV-1000, Olympus, Tokyo, Japan).

### 4.9. Statistical Analyses

Statistical analyses were performed using the Student’s two-tailed *t*. All analyses were performed using GraphPad Prism Software Version 6 (GraphPad Software Inc., San Diego, CA, USA). *p*-values < 0.05 were considered to be statically significant.

## Supplementary Materials

The following are available online at http://www.mdpi.com/1422-0067/19/4/1261/s1.

## Abbreviations

DAPI	4′,6-diamidino-2-phenylindole
DCs	Dendritic cells
ELISA	Enzyme-linked immunosorbent assay
FBS	Fetal bovine serum
IFN	Interferon
I.p.	Intraperitoneal
IL	Interleukin
LPS	Lipopolysaccharide
MDSCs	Myeloid-derived suppressor cells
MHC	Major histocompatibility complex
MRC1	Mannose receptor C-type 1
NK	Natural killer
RPMI	Roswell park memorial institute
S.c.	Subcutaneously
TAM	Tumor-associated macrophages
TLRs	Toll-like receptors
TNF-α	Tumor necrosis factor-α
WT	Wild type
