# Supplementary material for: Cytokine Expression and Macrophage Localization in Xenograft and Allograft Tumor Models Stimulated with Lipopolysaccharide
Source: Int J Mol Sci. 2018 Apr 23;19(4):1261. doi: 10.3390/ijms19041261 (PMC5979423; doi:10.3390/ijms19041261)
Supplement: Supplementary file 1 [file ijms-19-01261-s001.pdf]

International Journal of
Molecular Sciences

MDPI

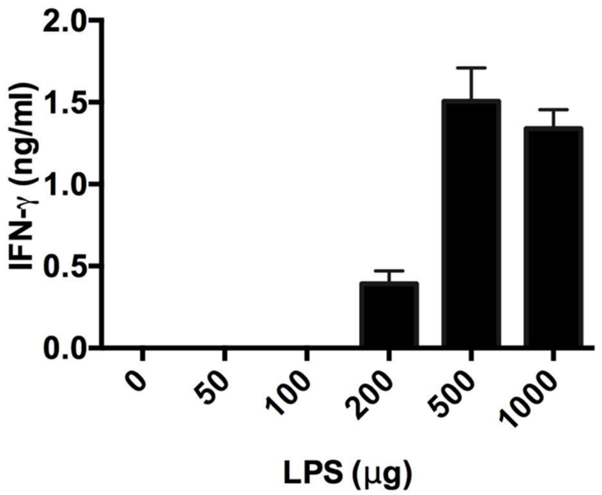

Figure S1. IFN-γ production by LPS in nude mice. A serial concentration of LPS (0, 50, 100, 200, 500, 1000 μg in 200 μL PBS /body) was i.p. injected and blood samplings were carried out at 6 h. The serum levels of IFN-γ were determined by ELISA. The data are presented as the mean ± SEM. $n = 5$.
